# Rickets

**Published:** 1910-03-12

**Authors:** Edmund Cautley

**Affiliations:** 15 Upper Brook Street, W.


### Editor's Letter-Box.

RICKETS.

*To the Editor of* The Hospital.

I have noticed in an article on enlargement of the liver, published in your paper on February 19, the following statement, which is worthy of criticism. The writer states that "In the early stages of the disease there is general tenderness of the whole body, particularly along the bones, slight pyrexia, restlessness at night, fatness, and overweight; unless intercurrent maladies, such as diarrhœa and vomiting, or bronchitis and broncho-pneumonia, lead to marasmus, great restlessness and profuse sweating about the head and neck at night."

I doubt whether such statements should be so dogmatically expressed in a journal with a large circulation. There is no reliable evidence that rickets produces tenderness of the bones. Such tenderness, when present, is practically invariably due to scurvy, which may or may not be associated with the disease. Pyrexia is not an early sign of rickets, but is due to an associated factor, a complication. Restlessness at night occurs from many causes. Fatness and overweight are not early stages of rickets, inasmuch as they may be independent of this disease, and rickets can occur in the thin and ill-nourished. It is difficult to elucidate the meaning of the second half of the sentence quoted. Does the writer mean that "great restlessness and profuse sweating" are due to rickets or to intercurrent maladies? He states that the early stages of rickets include restlessness at night" unless intercurrent maladies . . . lead to marasmus, restlessness, and profuse sweating . . . at night." The vagueness of the whole sentence is of less importance than the repetition of the exploded notion that tenderness is a sign of rickets. Such teaching inevitably leads to the erroneous diagnosis of scurvy as a rachitic phenomenon in its early stages, at a time when it readily responds to efficient treatment.

I remain, yours faithfully,

Edmund Cautley.

15 Upper Brook Street, W., March 6, 1910.

For those who can afford holiday at Easter time a sea trip towards the South has much to recommend it. The Orient Company offer a tempting opportunity in the shape of a trip to Gibraltar and Marseilles and back, an important feature of which is that the voyage each way is made in one of their new and palatial 12,000-ton twin-screw vessels. The *Orvieto* leaves London on March 18, and the return steamer, the *Osterley*, arrives back in London on April 1. Full particulars can be obtained on application to the Orient Line, Fenchurch Avenue, London, E.C.

